# Assessing the Sensitivity and Efficiency of Laser-Induced Breakdown Spectroscopy (LIBS) for High-Concentration Cadmium Detection in Cocoa Powder

**DOI:** 10.3390/s25082434

**Published:** 2025-04-12

**Authors:** Juan Molina M., Raquel Pincay, Víctor Santos, José Luis González, María Fernanda Trujillo Guerrero, Diego Díaz Pace, César Costa-Vera

**Affiliations:** 1 Consejo Nacional de Investigaciones Científicas y Técnicas (CONICET), Calle Godoy Cruz 2290, Buenos Aires 1425, Argentina; ddiaz@ifas.exa.unicen.edu.ar; 2 Laboratorio de Ecología Evolutiva Humana (LEEH), Facultad de Ciencias Sociales (FACSO), Universidad Nacional del Centro de la Provincia de Buenos Aires (UNICEN), Calle 508 881, Quequén, Buenos Aires 7631, Argentina; 3 Mass Spectrometry & Optical Spectroscopy Group, Departamento de Física, Escuela Politécnica Nacional (EPN), Ladrón de Guevara E11–253, Ed. #6, Piso 1, Quito 170525, Ecuadorcesar.costa@epn.edu.ec (C.C.-V.); 4 Departamento de Automatización y Control Industrial, Escuela Politécnica Nacional, Ladrón de Guevara E11-253, Quito 170525, Ecuador; victor.santos@epn.edu.ec (V.S.); maria.trujillo01@epn.edu.ec (M.F.T.G.)

**Keywords:** laser-induced breakdown spectroscopy, LIBS, cadmium, cocoa

## Abstract

**Highlights:**

**What are the main findings?**

- The LIBS technique was successfully applied for the first time to quantify cadmium in commercial cocoa powder across concentrations ranging from 70 ppm to 5000 ppm.
- A mechanical mixing and pelletization protocol for sample preparation, combined with a background correction algorithm, significantly improved the accuracy and robustness of LIBS for cadmium detection in complex cocoa matrices.

**What are the implications of the main findings?**

- LIBS demonstrates its potential as a rapid, field-deployable method for food safety monitoring and industrial contamination assessments.
- The methodology validates its applicability in high-contamination scenarios and expands the use of LIBS to other plant-based matrices and environmental monitoring.

**Abstract:**

Cocoa is a major commodity in the global food industry. Heavy metal contamination, particularly cadmium (Cd), raises significant concerns. This work demonstrates the use of laser-induced breakdown spectroscopy (LIBS) for fast Cd quantification in commercial cocoa powder across a wide range of concentrations (70–5000 ppm). Cocoa powder presents unique challenges due to its physical properties, such as the tendency to soften and liquefy at elevated temperatures, which complicates sample preparation. To address these issues, a mechanical mixing and pelletization protocol was implemented to ensure uniformity. Pellets were doped with known cadmium concentrations for calibration. Cadmium atomic lines at 340.36 and 361.05 nm were used to construct quantification curves. A special algorithm for background subtraction was implemented, and the LIBS plasma was characterized to ensure local thermodynamic equilibrium conditions. Out of eighteen samples, five double-blinded unknowns were evaluated. The concentrations agreed well within normalized standard deviations of 9.73% and 5.88% for the two cadmium lines. The limits of detection for the lines were 0.4 and 0.08 μg/g, respectively. LIBS is confirmed as a rapid and versatile analytical tool for Cd detection and quantification in complex food matrices, with potential applications in field-based and industrial monitoring systems.

## 1. Introduction

Cocoa is a rich source of bioactive compounds, fats, carbohydrates, proteins, antioxidants, and essential minerals. Beyond its nutritional value, it plays a crucial economic and cultural role, particularly in Latin America. The domestication of cacao is believed to have occurred over 5000 years ago, either in Central America or the Amazon basin, particularly in southeastern Ecuador [1]. Historically, cacao was highly valued by ancient civilizations such as the Mayans, Aztecs, and the Andean cultures of Valdivia and Machalilla in Ecuador, as well as Puerto Hormiga and San Agustín in Colombia, where it was used both as currency and a trade item [2]. Today, Ecuador remains one of the world’s leading cocoa exporters, contributing approximately 6% of global cocoa production [3,4].

Heavy metal contamination in foods, particularly cadmium (Cd), raises significant concerns. Cd contamination originates from natural sources such as soil and water, as well as anthropogenic activities, posing serious health risks due to its bioaccumulation and toxicity. Regulatory bodies, such as the European Union, have established stringent limits for Cd in cocoa products, starting at 0.1 and rising to 0.8 ppm, depending on the product category [5,6]. These limits aim to safeguard consumers but present challenges for producers in regions with naturally elevated Cd levels in the soil, especially in remote locations.

Detecting Cd at regulatory levels is crucial, but evaluating analytical methods capable of performing beyond these thresholds is equally important to assess their robustness and sensitivity. This study investigates the use of laser-induced breakdown spectroscopy (LIBS) for quantifying Cd in cocoa powder across a wide range of concentrations, from 70 ppm to 5000 ppm. Although the lower end of this range exceeds typical dietary exposure levels, it validates LIBS as a powerful tool for analyzing complex matrices under diverse conditions, including scenarios of extreme contamination or industrial applications. This is a step toward implementing a general LIBS-based methodology usable at cacao bean plantations directly, where other more established methodologies cannot be applied in situ.

While the limit of detection (LoD) obtained in this study suggests that LIBS could be applied to cocoa samples with Cd concentrations at regulatory levels (0.1–0.8 ppm), our focus was on evaluating its performance under high-contamination conditions. The primary reason for not including sub-ppm samples was the challenge of achieving homogeneous sample preparation at such low concentrations. Future work should address these challenges by optimizing sample preparation techniques and calibration strategies to fully validate LIBS as a tool for detecting Cd at trace levels.

Several spectrometric techniques, such as atomic absorption spectroscopy (AAS), X-ray fluorescence spectroscopy (XRF), and atomic emission spectroscopy (AES), have been employed for detecting heavy metals in plants, seeds, and other matrices [7,8,9]. However, these techniques often require expensive equipment, highly specialized operators, and long processing times, making them less suitable or unapplicable for rapid or field-based analysis.

In contrast, LIBS emerges as an efficient alternative for identifying and quantifying cadmium in complex matrices like cocoa powder. This method uses a pulsed laser to generate a plasma that contains elemental information from the ablated sample surface. The unique composition of cocoa, including organic compounds such as fats and antioxidants, introduces a well-known matrix effect [10]. The interaction of organic compounds with laser-induced plasmas can lead to the formation and extinction of various chemical species, influenced by irradiation parameters [11]. Addressing these challenges requires robust sample preparation techniques, precise background correction algorithms, and plasma characterization to ensure accurate quantification [12,13].

Gamela et al. [10] identified this matrix-induced variability as a major limitation, while Moros and Laserna [14] highlighted the impact of spectral interferences and self-absorption in LIBS analysis of organic compounds. Additionally, Yang et al. [15] and Wang et al. [16] emphasized that sample preparation methods strongly influence the accuracy and reproducibility of LIBS-based measurements, particularly in complex food matrices.

This work aims to validate the LIBS technique for Cd quantification in cocoa powder, focusing on fast acquisition, sample preparation, data handling, calibration curve construction, and application in high-concentration scenarios. By testing this methodology on well-controlled, double-blind samples, we demonstrate its suitability not only for routine food safety monitoring, but also for industrial and environmental assessments involving elevated contamination levels.

## 2. Experimental

### 2.1. Sample Preparation

In this study, Pacari^®^ organic cocoa powder (Pacari Co., Quito, Ecuador) was used to prepare the samples. The content of a unique package of raw cocoa (100% Manabi variety) was homogenized mechanically and 1 g pellets were formed by compressing the powder into a stainless-steel die with a hydraulic press. A number of pellets (as explained below), including a reference sample (zero cadmium added) and pellets with specific amounts of tetrahydrate cadmium nitrate, Cd(NO_3_)_2_●4H_2_O (Sigma-Aldrich, Darmstadt, Germany 98%) [14], were produced.

Initially, as a base for all preparations, a total of 4.5000 g of tetrahydrate cadmium nitrate was dehydrated on a hot plate by gradually increasing the temperature from 150 °C to 300 °C to ensure complete evaporation of absorbed water in a contamination-free environment. The dehydrated remining salt (1.6095 g of the element cadmium) was then homogenized by pulverizing it in a mortar [15,17].

After homogenization of the Cd-doped salt, 1.7500 g of cocoa powder was added to the dried salt, forming a base solid mixture with a cadmium concentration of 9197 ppm. Eighteen samples were created by diluting different quantities of the base mixture with additional cocoa powder (to complete 1 g), resulting in Cd concentrations ranging from 70 to 5000 ppm to simulate varying contamination levels [14,15,16,17]. The doped samples resulted in cylindrical pellets with a diameter of 15.5 mm using a hydraulic press, achieving a uniform height of 2.90 mm after sanding [14]. This procedure ensures an optimized uniform distribution of analytes within the solid matrix [17].

The uncertainty in the cadmium concentration of the pellets arises from several factors, including the precision of the weighing process, the homogeneity of the mixture, and the repeatability of the pelletization procedure. The uncertainty in the added Cd concentration was estimated based on the standard deviation of multiple preparations using the same methodology. In this study, the overall uncertainty in the concentration of cadmium in the pellets was determined by evaluating the reproducibility of independent sample preparations, leading to relative uncertainties ranging between 4% and 15%.

### 2.2. Experimental Setup

**Figure 1:**
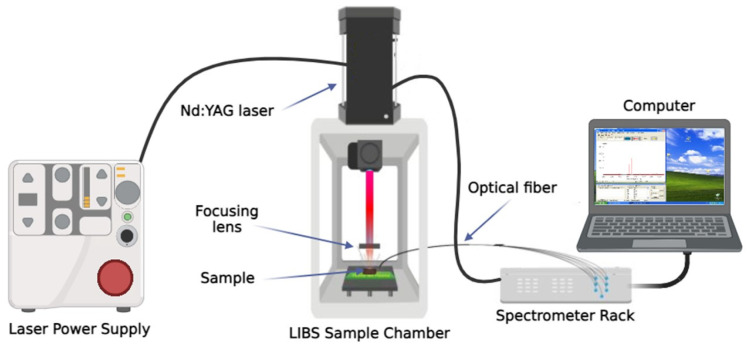
Experimental LIBS setup.

A schematic diagram of our LIBS system is shown in Figure 1. A LIBS 2000+ (Ocean Optics, Inc., Dunedin, FL, USA) was employed for this study. The system comprises a Nd:YAG laser (1064 nm, 8 ns, up to 250 mJ/pulse), seven concatenated high-resolution Czerny-Turner fiber optic spectrometers (Ocean Optics Inc, Dunedin, FL, USA, HR2000, 0.1 nm resolution), and a digital signal delay generator. Samples were mounted on a 2D, flat, displaceable platform, allowing precise selection of the laser impact point. A mobile convex lens with a focal length of 50 mm was used to focus the beam on the sample surface [16,17,18,19].

Measurements were performed under atmospheric pressure conditions with an air atmosphere, using a gate delay of 3 μs, a gate width of 10 μs, and an integration time of 1.05 ms. The laser energy was set to 75 mJ/pulse, and the lens-to-sample distance (LSTD) was 82 mm. Each pellet was irradiated with 10 laser shots per point, at a consecutive interval of 20 ms. The accumulated shots from 5 different positions on the pellet were collected to maintain fresh samples in each acquisition. These five accumulated spectra were further averaged, which has been shown to significantly enhance results by compensating for any slight inhomogeneity in the sample [20,21]. The delay time was optimized to enhance the signal-to-noise ratio for the selected Cd I emission lines at 340.36 nm and 361.05 nm while reducing spectral interference from matrix elements. Additionally, this delay time provided favorable conditions for the detection of Mg I and Mg II lines used in the Saha–Boltzmann plot for plasma temperature determination. Alternative Cd spectral lines were considered; however, many significantly overlapped with emissions from other elements in the cocoa matrix, making them impractical for this specific application.

In total, 18 pellets with different Cd concentrations were prepared. Spectra were recorded as indicated in each case. In a double-blind manner, 5 samples were selected and tested as unknowns to validate the methodology.

The proprietary OOILIBS software 5.1.0.0 (Ocean Optics Inc., Dunedin, FL, USA) was used for spectral acquisition and element identification through a spectral database for qualitative measurements. The identified emission lines were validated against the National Institute of Standards and Technology (NIST) database [22].

## 3. Results

### 3.1. Spectral Treatment

The data processing in this article was performed using Python 3.8^TM^. An algorithm for second and third local minima data fitting was used to preprocess the raw data, provided by the acquisition software. Further quantitative analysis was performed using Origin 2023 (OriginLab Co., Northampton, MA, USA).

As suggested above, variations in spectral intensities can be generated throughout the laser ablation process. Instrument parameters, the physical attributes of the sample, and other factors, such as reverse bremsstrahlung, recombination, electronic noise, environmental conditions, and the matrix effect contribute to this situation and may introduce outliers in the response [19,23,24]. Also, these factors contribute to the presence of different background levels, even among spectra within the same dataset [24,25]. The emission spectra were recorded with a delay time of 3 μs to minimize the influence of early continuum radiation and improve the signal-to-noise ratio. This introduced a significant amount of background in the spectra, which is detrimental to quantification. To improve the accuracy of the analysis and the efficiency of LIBS, the implementation of suitable methods for managing the spectrum and reducing the background is necessary. This ensures that the intensity of characteristic spectral lines in the LIBS spectrum remains consistent with the net intensity value corresponding to the concentration of Cd after this adjustment. [19,23].

#### Base Correction Algorithm

**Figure 2:**
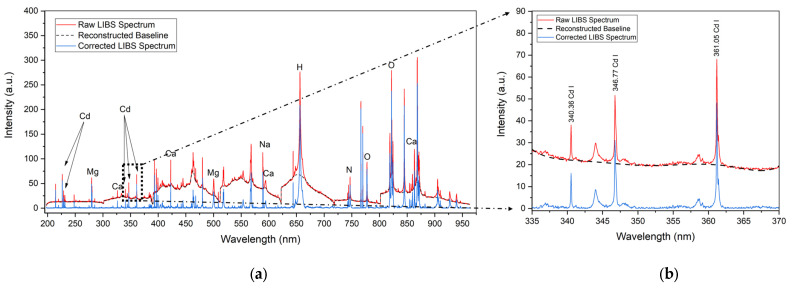
(**a**) Measured and spectral treatment LIBS all-range spectra from raw cocoa pellets (214.72 ppm concentration); (**b**) the cadmium interest spectral section used in this work. The red spectra have a strong background component due to electron reverse bremsstrahlung radiation. This component is efficiently removed with our algorithm.

A recurrent algorithm in LIBS spectral treatment, applied to both real and simulated spectra—both simple and complex, as illustrated in Figure 2—is based on the identification of numerous local minima combined with background interference signals.

In LIBS spectral analysis, recurring algorithms are commonly used to process both direct and simulated spectra, ranging from straightforward to more intricate cases. Many of these algorithms rely on the piecewise identification of multiple local minima, considering the nature of the background signal. This approach allows for accurate baseline tracking and effective removal of background interference while preserving relevant spectral information in the characteristic emission lines [25,26,27,28,29].

We have introduced an algorithm based on the method proposed by Liu et al. [25]. The raw LIBS spectrum was divided into sections corresponding to each of the 7 daisy-chained spectrometers to cover the full spectral range, and each section was analyzed to identify n third-order minima and their intensities, along with m adjacent second-order minima with their respective intensities. The spectra were then segmented into n+1 consecutive sub-intervals using third-order minima as reference points. The mean intensity of the second-order minima in each subinterval served as a threshold for filtering out background contributions. Next, second-order minima in each subinterval were compared to their respective thresholds, eliminating points that exceeded the threshold intensity. To mitigate excessive point distances, the set of effective points (filtered second-order minima) was expanded through linear interpolation. A higher-order polynomial was used to approximate the background interference signal profile, with the polynomial order chosen between 1 and 10 through a least-squares criterion. The polynomial order (k) for each segment was selected by minimizing the chi-squared value of the fit. The standard deviation with extension points was determined using the mean square error. Finally, the reconstructed baseline of the background interference signal was subtracted from the original spectrum, minimizing background noise intensity and avoiding spectral distortion [20]. As indicated, this algorithm was applied separately to each spectrometer to account for inherent differences in spectral resolution and sensitivity, which can manifest as unique background patterns due to electronic noise and reverse bremsstrahlung effects [26,27,28,29].

To improve background correction in LIBS spectra for organic matrices, we adapted the algorithm described in [25] by introducing a dynamic baseline selection method, polynomial fitting adjustments to mitigate matrix effects, and an optimized filtering approach to reduce over-correction artifacts. While the original algorithm in [25] was designed for general spectral applications, our approach specifically addresses the challenges of LIBS spectra in organic samples such as cocoa powder, where variable baselines and matrix-induced spectral interferences are common. These modifications enhance the robustness of background subtraction, ensuring more accurate spectral analysis and improving the reproducibility of LIBS measurements in complex matrices.

### 3.2. LIBS Analysis

Following spectral treatment, the spectra were analyzed to identify the detected Cd spectral lines. Spectral lines exhibiting a suitable signal-to-noise ratio, moderate self-absorption, a low limit of detection, and minimal spectral interferences from other elements present in cocoa beans were selected as Cd analytical lines (340.36 and 361.05 nm). These analytical lines correspond to transitions associated with neutral atomic species, for which atomic data were available from the NIST database, as summarized in Table 1.

While alternative non-resonant emission lines could help reduce self-absorption effects, the traditionally used Cd transitions overlap with emissions from other elements in the cocoa matrix, limiting their applicability.

The intensities of the spectral lines listed in Table 1, corresponding to each concentration in the selected calibration curve pellets, were measured following spectral processing. The Cd I lines at 340.36 nm and 361.05 nm were analyzed using a Voigt function to account for both Stark and instrumental broadening, ensuring reliable peak integration despite the spectrometer’s resolution limitations. The integrated areas of the line profiles were quantified, and these net intensities were compiled and subjected to fitting procedures to construct the calibration curve.

#### 3.2.1. Plasma Characterization

**Figure 3:**
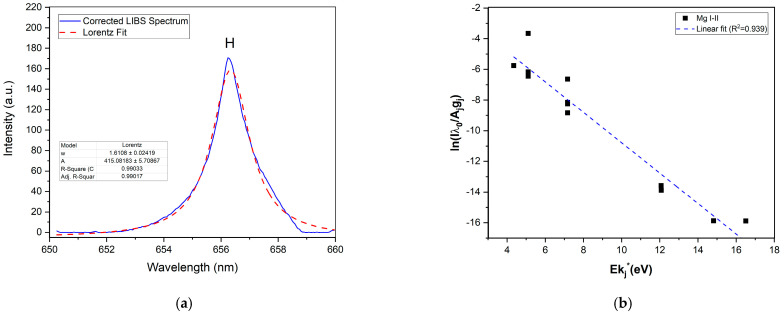
(**a**) The 656.3 nm $H_{\alpha }$ hydrogen line is shown together with the corresponding curve fitting with $w_{L}=1.6$ nm; (**b**) the Saha–Boltzmann plot constructed using integrated Mg I and II atomic and ionic lines. The horizontal axis represents the excitation energy of the upper levels of the observed transitions (eV), while the vertical axis represents the logarithm of the intensity ratio. The plasma temperature is determined from the slope of the linear fit, which accounts for these intensities and their corresponding energy level gaps [20].

The determination of plasma parameters was crucial to ensure that the measurements were made under local thermodynamic equilibrium (LTE) conditions [31,32], a prerequisite for accurate quantitative analysis. In this study, on the one hand, the electronic density of the LIBS plasma was estimated by fitting the 656.3 nm $H_{\alpha }$ line with a Lorentzian function, as shown in Figure 3. With the Lorentzian width, $w_{L}$, the mean electronic density (*N_e_*) can be obtained using the following equation [33]:

(1) $$H_{\alpha }:N_{e}[m^{-3}]=10^{23}{\left(\frac{w_{L}[nm]}{1.098}\right)}^{1.47}.$$

On the other hand, to determine the mean temperature of the plasma, the Mg I–II emission lines were fitted to Voigt profiles [34,35], and a Saha–Boltzmann plot was constructed using the intensities of both species. This assumption is grounded in the typical fulfillment of LTE conditions in LIBS experiments, attributed to the relatively high electron densities in the plasma [32]. The spectroscopic parameters for the Mg lines are presented in Table 2.

With the estimated mean electron density *N_e_* = (1.7 ± 0.1) 10^17^ ${cm}^{-3}$, the mean temperature of the plasma, *T* = 15230 ± 47 K (1.31 eV), was determined from a Saha–Boltzmann plot (Figure 3, ref. [20]) constructed using the net emission intensities of the Mg I–II lines. The calculated electron density and plasma temperature align with values typically observed in LIBS applications, confirming the robustness of the measurements. In order to guarantee that the spectra were obtained under LTE-conditions, the McWhirter criterion was evaluated [32].

(2) $$N_{e}\geq 1.6\times 10^{12}{\left(T\right)}^{\frac{1}{2}}{\left(∆E\right)}^{3},$$

where Δ*E* is the energy difference (in eV) between the upper and lower cadmium energy levels in the radiative transition used for electron density estimation. For this work, $N_{e}=$ 1.7 × 10^17^ ${cm}^{-3}\geq$ 7.6 × 10^16^ ${cm}^{-3}$, and thus, McWhirter’s criterion is satisfactorily met.

#### 3.2.2. Calibration Curve and Detection Limits

**Figure 4:**
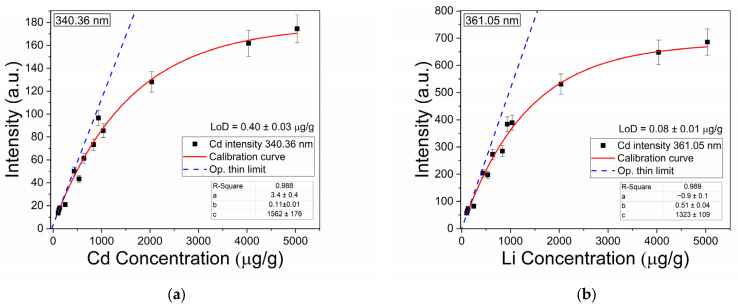
Calibration curves used to estimate the unknown concentrations of cadmium obtained for lines (**a**) 340.36 nm Cd I and (**b**) 361.05 nm Cd I.

The Cd concentration calibration curve was constructed from 340.36 nm and 361.05 nm intensities of the cocoa samples acquired plotted against concentration, as depicted in Figure 4. Each data point represents the average of 50 measurements taken on each sample. Here, the error bars correspond to the relative standard deviation, ranging between 2% and 10%, which is consistent with values in the literature [34,36].

To obtain an analytical form for the predictive calibration curve, a fit to an exponential equation with a saturated region and a non-linear function was used [27]. This enhanced the accuracy of the calibration curves, particularly for high-concentration scenarios relevant to industrial monitoring.

(3) $$y=a+bc(1-e^{-x/c})$$

In this equation, *x* symbolizes concentration while *y* denotes intensity, and the constant *c* represents the concentration at which self-absorption becomes representative. The obtained determination coefficient (*R*^2^ = 0.998) confirms the reliability of the model for quantifying Cd in cocoa powder samples. The linear fitting followed the equation

(4) y=a+bx,

which is valid under optically thin plasma conditions. Parameter *b* can be employed to compute the detection limit. In both cases, self-absorption was detected at concentrations above 1500 μg/g.

For the detection limit of Cd, the linear section of the calibration curve was used (blue dashed line), following the expression proposed in [37],

(5) $$LoD=\frac{3\sigma }{s},$$

where σ is the standard deviation of the noise at the wings of the intensity peak and *s* is the sensitivity given by the slope of the calibration curve. The corresponding limits of detection calculated for the 340.36 nm Cd I and 361.05 nm Cd I lines were 0.40 ± 0.03 μg/g and 0.08 ± 0.01 μg/g, respectively. Interestingly, both detection limits are in the order of the concentration of cadmium present in Ecuadorian and other origin cocoas [38].

**Figure 5:**
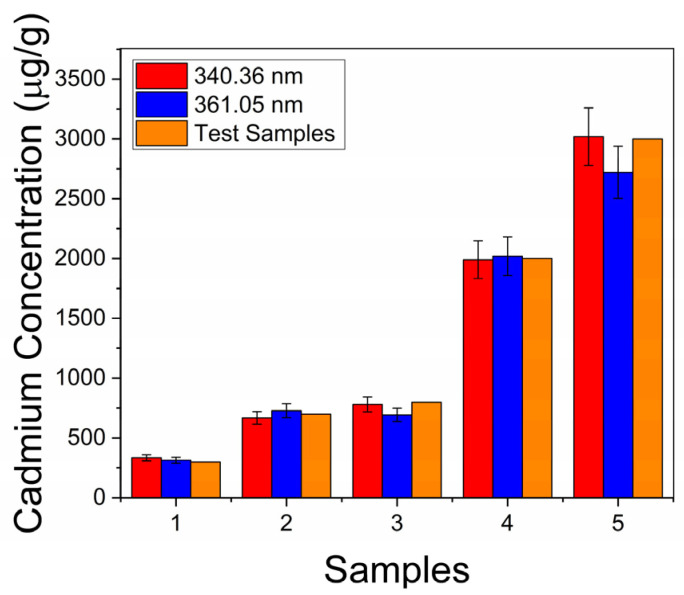
Comparison of Cd concentration in cocoa samples. The orange bars correspond to the mixed-in concentrations in the test samples, and the red and blue bars show the corresponding concentrations derived from the calibration curves for the 340.36 and 361.05 nm lines. Very good agreement is found between the three cases.

As mentioned before, we selected five samples in a double-blind manner to be used as unknowns and used the calibration curve to determine their cadmium content in order to validate the LIBS methodology we are introducing. This is shown in Table 3 and Figure 5.

**Figure 6:**
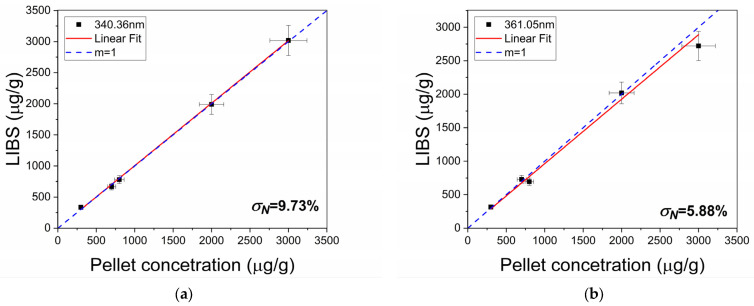
Determination of the corresponding Cd concentration in the cocoa test samples from the LIBS calibration curves including saturation, in (**a**) the 340.36nm Cd I line, and (**b**) the 361.05nm Cd I line. The error bars are at scale and are, as expected, larger in the saturation region due to diminishing sensibility. The dashed *m* = 1 line represents the ideal case where the measured LIBS concentration matches the actual pellet concentration.

To assess the accuracy of our LIBS results, the averaged quantification error between the concentration of (unknown) test samples and the concentration obtained by the calibration curve, as shown in Figure 6, was evaluated using a normalized standard deviation.

(6) $$\sigma_{N}=\sqrt{\frac{\sum {\left(\frac{q_{LIBS}-q_{AAS}}{q_{LIBS}}\right)}^{2}}{n-1}}\cdot 100,$$

Values of $\sigma_{N}=9.73\%$ and $\sigma_{N}=5.88\%$ were obtained for the 340.36 nm and the 361.05 nm spectral lines, respectively. This demonstrates the robustness and reliability of the proposed LIBS method.

## 4. Conclusions

In this work, the LIBS technique was successfully applied for the rapid and quantitative analysis of cadmium in consumer-ready commercial cocoa powders across a wide concentration range. By addressing concentrations beyond regulatory levels, this study demonstrates the robustness and adaptability of fast LIBS analysis for complex matrices, including applications in industrial and environmental monitoring. The methodology integrated an optimized sample preparation process, double-blind sampling, a distinct background removal algorithm, and accurate calibration curve construction. The results confirm LIBS to be a rapid, robust, and complementary alternative to traditional spectroscopic techniques. With a low detection limit (<1 µg/g), the method is feasible for compliance with cadmium regulations in human consumption and industrial settings. Furthermore, it efficiently quantifies high cadmium concentrations (around 2000 µg/g), broadening its scope of application.

The use of compact lasers and spectrometers in this study highlights the potential of LIBS for fast, on-site cadmium quantification, which we aim to implement next. Future work could explore its application beyond heavy metal detection, such as analyzing protein and fat content, to further expand its utility in cocoa quality control and in different components of the environment surrounding cacao plants—such as soil, water, plant structures, and other matrices—to benefit from its agility, and versatility, and to expand its applicability to other important analytical challenges.

## Figures and Tables

**Table 1:** Cd lines used for plasma characterization [30].

Element	Ionization State	Wavelength (nm)	$A_{ji}(s^{-1})$	$E_{j}(eV)$	$E_{i}(eV)$	$g_{j}$	$g_{i}$
Cd	I	340.36	7.07 × 10^7^	3.73	7.36	0	1
Cd	I	361.05	1.30 × 10^8^	3.94	7.38	2	3

**Table 2:** Mg I-II lines used for plasma characterization [31].

Species	Wavelength (Å)	$A_{ji}({10^{8}s}^{-1})$	$E_{j}(eV)$	$E_{i}(eV)$	$g_{j}$	$g_{i}$
Mg I	2776.47	1.32	2.712	7.175	3	5
Mg I	2778.45	1.82	2.709	7.170	1	3
Mg I	2779.65	1.36	2.712	7.170	3	3
Mg I	2781.00	5.43	2.717	7.173	5	3
Mg I	2782.78	2.14	2.717	7.170	5	3
Mg I	2852.14	4.91	0.000	4.345	1	3
Mg I	5167.09	1.13	2.709	5.107	1	3
Mg I	5172.59	3.37	2.712	5.108	3	3
Mg I	5183.53	5.61	2.717	5.108	5	3
Mg II	2790.78	4.01	4.422	8.864	2	4
Mg II	2795.23	2.60	0.000	4.434	2	4
Mg II	2798.25	4.79	4.434	8.863	4	6
Mg II	2802.50	2.57	0.000	4.422	2	2

**Table 3:** Estimated Cd concentrations of the unknowns (test samples) evaluated in this work (LIBS).

Test Samples Mixed-in Concentration (μg/g)	Calculated Concentration (μg/g)	
	Cadmium Lines	
	361.05 nm	340.43 nm
300	314 ± 25	335 ± 26
700	728 ± 58	668 ± 53
800	728 ± 55	780 ± 62
2000	2019 ± 161	1990 ± 159
3000	2720 ± 217	3018 ± 241

## Data Availability

Data analyzed in this manuscript may be shared upon reasonable request.

## References

[1] Zarrillo S., Gaikwad N., Lanaud C., Powis T., Viot C., Lesur I., Fouet O., Argout X., Guichoux E., Salin F. (2018). The use and domestication of *Theobroma cacao* during the mid-Holocene in the upper Amazon. Nat. Ecol. Evol..

[2] Lanaud C., Vignes H., Utge J., Valette G., Rhoné B., Garcia Caputi M., Angarita Nieto N.S., Fouet O., Gaikwad N., Zarrillo S. (2024). A revisited history of cacao domestication in pre-Columbian times revealed by archaeogenomic approaches. Sci. Rep..

[3] Lippi D., Vannozzi G. (2019). Cacao bioactive compounds: Chemistry, analysis, and bioavailability. Chocolate in Health and Nutrition.

[4] International Cocoa Organization (ICCO) (2022). Quarterly Bulletin of Cocoa Statistics, Volume XLVIII, No. 1–4. https://www.icco.org/categorie-produit/qbcs/.

[5] (1995). General Standard for Contaminants and Toxins in Food and Feed.

[6] European Commission (2006). Regulation (EC) No 1881/2006 of 19 December 2006 setting maximum levels for certain contaminants in foodstuffs. Off. J. Eur. Union.

[7] Smith R.M., Martell A.E. (1976). Critical evaluation of selected atomic absorption techniques for the determination of metals in plants. Anal. Chem..

[8] Marguí E., Queralt I., Hidalgo M. (2009). Application of X-ray fluorescence spectrometry to determination and quantitation of metals in vegetal material. TrAC Trends Anal. Chem..

[9] Zhong W.-S., Ren T., Zhao L.-J. (2016). Determination of Pb (Lead), Cd (Cadmium), Cr (Chromium), Cu (Copper), and Ni (Nickel) in Chinese tea with high-resolution continuum source graphite furnace atomic absorption spectrometry. J. Food Drug Anal..

[10] Gamela R.R., Costa V.C., Babos D.V., Araújo A.S., Pereira-Filho E.R. (2020). Direct determination of Ca, K, and Mg in cocoa beans by laser-induced breakdown spectroscopy (LIBS): Evaluation of three univariate calibration strategies for matrix matching. Food Anal. Methods.

[11] Moros J., Laserna J.J. (2019). Laser-induced breakdown spectroscopy (LIBS) of organic compounds: A review. Appl. Spectrosc..

[12] Douglas A.S., Holler F.J., Crouch S.R. (2013). Fundamentals of Analytical Chemistry.

[13] Jantzi S.C., Motto-Ros V., Trichard F., Markushin Y., Melikechi N., De Giacomo A. (2016). Sample treatment and preparation for laser-induced breakdown spectroscopy. Spectrochim. Acta Part B At. Spectrosc..

[14] Fu X., Li G., Tian H., Dong D. (2018). Detection of cadmium in soils using laser-induced breakdown spectroscopy combined with spatial confinement and resin enrichment. RSC Adv..

[15] Yang P., Zhou R., Zhang W., Yi R., Tang S., Guo L., Hao Z., Li X., Lu Y., Zeng X. (2019). High-sensitivity determination of cadmium and lead in rice using laser-induced breakdown spectroscopy. Food Chem..

[16] Wang W., Kong W., Shen T., Man Z., Zhu W., He Y., Liu F., Liu Y. (2020). Application of laser-induced breakdown spectroscopy in detecting cadmium content in rice stems. Front. Plant Sci..

[17] Díaz Pace D.M., D’Angelo C.A., Bertuccelli D., Bertuccelli G. (2006). Analysis of heavy metals in liquids using laser-induced breakdown spectroscopy by liquid-to-solid matrix conversion. Spectrochim. Acta Part B.

[18] Peng J., He Y., Zhao Z., Jiang J., Zhou F., Liu F., Shen T. (2019). Fast visualization of chromium in rice leaves using dual-pulse LIBS and chemometric methods. Environ. Pollut..

[19] Gondal M.A., Hussain T., Yamani Z.H., Baig M.A. (2006). Detection of heavy metals in Arabian crude oil residue using LIBS. Talanta.

[20] Tognoni E., Palleschi V., Corsi M., Cristoforetti G., Omenetto N., Gornushkin I., Smith B.W., Winefordner J.D. (2006). From Sample to Signal in laser-Induced Breakdown Spectroscopy: A Complex Route to Quantitative Analysis. Laser-Induced Breakdown Spectroscopy (LIBS) Fundamentals and Applications.

[21] El Haddad J., Canioni L., Bousquet B. (2014). Good practices in LIBS analysis: Review and advice. Spectrochim. Acta Part B.

[22] Kramida A., Ralchenko Y., Reader J., NIST ASD Team NIST Atomic Spectra Database (Ver. 5.10). https://physics.nist.gov/asd.

[23] Dyar M.D., Giguere S., Carey C.J., Boucher T. (2016). Comparison of baseline removal methods for laser-induced breakdown spectroscopy of geological samples. Spectrochim. Acta Part B.

[24] Giguere S., Boucher T., Carey C.J., Mahadevan S., Dyar M.D. (2017). A fully customized baseline removal framework for spectroscopic applications. Appl. Spectrosc..

[25] Liu J., Zhang R., Li X., Chen J., Liu J., Qiu J., Gao X., Cui J., Heshig B. (2018). Continuous background correction using effective points selected in third-order minima segments in low-cost laser-induced breakdown spectroscopy without intensified CCD. Opt. Express.

[26] Palleschi V. (2023). Chemometrics and Numerical Methods in LIBS.

[27] Cremers D.A., Radziemski L.J. (2013). Handbook of Laser-Induced Breakdown Spectroscopy.

[28] Noll R. (2012). Laser-Induced Breakdown Spectroscopy.

[29] Singh J.P., Thakur S.N. (2020). Laser-Induced Breakdown Spectroscopy.

[30] National Institute of Standards and Technology (NIST) Atomic Spectra Database. http://physics.nist.gov/PhysRefData.

[31] Cristoforetti G., Lorenzetti G., Palleschi V., Capitelli M. (2010). Local Thermodynamic Equilibrium in Laser-Induced Breakdown Spectroscopy: Beyond the McWhirter Criterion. Spectrochim. Acta Part B.

[32] Aragón C., Aguilera J.A. (2008). Characterization of laser-induced plasmas by optical emission spectroscopy: A review of experiments and methods. Spectrochim. Acta Part B.

[33] Konjević N., Ivković M., Sakan N. (2012). Hydrogen Balmer lines for low electron number density plasma diagnostics. Spectrochim. Acta Part B.

[34] Mansour S.A.M. (2015). Self-Absorption Effects on Electron Temperature Measurements Utilizing Laser-Induced Breakdown Spectroscopy (LIBS) Techniques. Opt. Photonics J..

[35] Diaz Pace D.M., Molina M.J. (2023). τ-algorithm for gathering spectroscopic information by modeling emission line shapes: Application to laser-induced plasmas. J. Opt. Soc. Am. B.

[36] Aragón C., Aguilera J.A., Pen F., Alba Á. (1999). Improvements in Quantitative Analysis of Steel Composition by Laser-Induced Breakdown Spectroscopy at Atmospheric Pressure Using an Infrared Nd:YAG Laser. Appl. Spectrosc..

[37] Sabsabi M., Cielo P. (1995). Quantitative Analysis of Aluminum Alloys by Laser-Induced Breakdown Spectroscopy and Plasma Characterization. Spectrochim. Acta Part B.

[38] Argüello D., Chávez E., Montalvo D. (2019). Soil Properties and Agronomic Factors Affecting Cadmium Concentrations in Cacao Beans: A Nationwide Survey in Ecuador. Sci. Total Environ..

